# Predicting sport fish mercury contamination in heavily managed reservoirs: Implications for human and ecological health

**DOI:** 10.1371/journal.pone.0285890

**Published:** 2023-08-22

**Authors:** Jesse M. Lepak, Brett M. Johnson, Mevin B. Hooten, Brian A. Wolff, Adam G. Hansen

**Affiliations:** 1 Colorado Parks and Wildlife, Fort Collins, CO, United States of America; 2 Department of Fish, Wildlife and Conservation Biology, Colorado State University, Fort Collins, CO, United States of America; 3 Department of Statistics and Data Sciences, College of Natural Sciences, The University of Texas at Austin, Austin, TX, United States of America; Indian Institute of Technology Hyderabad, INDIA

## Abstract

Mercury (Hg) is a concerning contaminant due to its widespread distribution and tendency to accumulate to harmful concentrations in biota. We used a machine learning approach called random forest (RF) to test for different predictors of Hg concentrations in three species of Colorado reservoir sport fish. The RF approach indicated that the best predictors of 864 mm northern pike (*Esox lucius*) Hg concentrations were covariates related to salmonid stocking in each study system, while system-specific metrics related to productivity and forage base were the best predictors of Hg concentrations of 381 mm smallmouth bass (*Micropterus dolomieu*), and walleye (*Sander vitreus*). Protecting human and ecological health from Hg contamination requires an understanding of fish Hg concentrations and variability across the landscape and through time. The RF approach could be applied to identify potential areas/systems of concern, and predict whether sport fish Hg concentrations may change as a result of a variety of factors to help prioritize, focus, and streamline monitoring efforts to effectively and efficiently inform human and ecological health.

## Introduction

Mercury (Hg) is a concerning contaminant due to its widespread distribution and tendency to bioaccumulate in organisms to concentrations that negatively influence the health of humans, other organisms, and ecosystems worldwide [1–3]. Mercury enters the landscape through a variety of natural and anthropogenic pathways including volcanoes, forest fires, erosion, fossil fuel burning, waste incineration, mining operations, and cement production [4]. Despite health concerns related to Hg contamination in fish, documented benefits of fish consumption may outweigh the risks [2,5,6], and healthy fish consumption has been encouraged [7]. Thus, understanding fish Hg concentrations and monitoring programs are necessary to protect human health related to fish consumption.

Sport fish Hg concentrations are generally a preferred indicator of Hg contamination in freshwater systems versus other aquatic organisms. Consumption of contaminated sport fish (with Hg incorporated in their muscle tissue) is the major method of transfer of aquatic Hg to humans [1,2]. However, fish Hg concentrations can be inherently variable due to several factors, and changes in contributions of Hg to the environment do not necessarily translate directly to changes in fish Hg concentrations because of complex biogeochemical interactions [8,9]. Thus, Hg deposition information alone cannot be used to infer fish Hg concentrations across the landscape, and extensive, large-scale monitoring programs can be cost prohibitive. Resource limitations within agencies, variability in fish Hg concentrations, and complex environmental interactions, make predictive model development (to identify priority areas or species of concern for testing) challenging. Approaches incorporating information about mechanisms influencing fish Hg concentrations and multiple data sources (including correlated predictors) could help overcome some of the obstacles to characterizing dynamic uncertainty in fish Hg concentrations.

Investigators have studied a variety of factors associated with Hg contaminated sport fish in North America [10–12]. Several patterns have emerged on the larger landscape. For example, when deposited, Hg can be taken up quickly by water and the organisms within it [13]. Because of this direct water-atmosphere interface, larger systems (higher surface area) receive more overall Hg deposition relative to smaller systems. In addition, systems in close proximity (<100 km) to “large” sources of Hg (similar to those found in the eastern and central United States) can experience increased Hg deposition [14]. Wentz et al. [15] found that Hg inputs to stream fishes were highest in urban versus rural areas. It follows that systems located near Hg sources (i.e., population centers or urban areas versus rural areas) could experience higher levels of Hg inputs and exposure.

Fish species, size, and trophic position are important factors influencing sport fish Hg concentrations [16–18]. Specifically, large, piscivorous sport fish with elevated trophic positions tend to have higher Hg concentrations than small, omnivorous or planktivorous fish feeding at lower trophic levels. Therefore, changes in food web structure (especially the forage base of sport fish) can alter sport fish Hg concentrations by changing fish trophic position, growth, and diet [19–21]. Aquatic food web structure and species interactions are often influenced by fisheries management practices (e.g., harvest regulations, fish stocking). Thus, these potential sources of variability are important to consider.

In the arid portion of western North America, artificial reservoirs are the predominant lacustrine systems on the landscape, and tend to be highly managed with respect to their biotic and abiotic characteristics. These characteristics can influence Hg dynamics [22], and several studies have found that reservoirs have fish and other biota that can be particularly high in Hg concentrations in general [23–25]. Reservoirs can experience changes in water levels, and water level fluctuation has been associated with elevated Hg concentrations in sport fish [26,27]. These studies suggest that fish Hg levels are influenced by water level fluctuation through the rewetting and perturbation of dry soils which are relatively rich in sulfate, stimulating population growth of sulfate-reducing bacteria, Hg methylation rates, and Hg uptake by biota. Analogous patterns of elevated Hg concentrations in biota (zooplankton and fish) in systems experiencing water level fluctuation have also been found [28].

Nutrient inputs also influence system productivity and subsequent Hg concentrations in sport fish. Increased productivity can reduce Hg concentrations in organisms through “bloom “dilution [29,30] or “growth dilution” [31]. When high-quality diet items are available to organisms, they typically display high rates of somatic growth paired with lower consumption rates. This reduces Hg concentration in prey and overall intake by predators. In the case of fish, it has been shown through experimental nutrient additions and observational studies that fish from systems with higher nutrient inputs have relatively low Hg concentrations [32–34]. Thus, increased nutrient inputs to reservoirs can reduce Hg bioaccumulation in sport fish within them. At the same time, Hg is methylated (becoming bioavailable) under anoxic conditions by bacteria as a byproduct of their energy sequestration pathway [35,36]. High nutrient inputs can stimulate primary production and decay creating anoxic conditions conducive to Hg methylation [37,38]. Hypoxic conditions have been associated with high concentrations of Hg in water, zooplankton, and fish [11,39].

Several additional factors can act as drivers that influence fish Hg concentrations depending on environmental or other conditions. For example, water temperature can influence growing season length (and subsequently growth dilution), community composition, and Hg methylation rates. All else being equal, fish that grow faster can be “diluted” in Hg relative to others growing more slowly [40]. Thus, more growth and longer growing seasons can result in lower fish Hg concentrations. However, warmer water may also stimulate microbial activity and Hg methylation, making Hg more available for uptake by biota. Therefore, elevation and latitude could influence sport fish Hg concentrations positively or negatively as they relate to differences in system-specific temperature regimes.

We describe a machine learning approach to identify predictors of Hg concentrations in three different sport fish species in Colorado, USA reservoirs. We use data compiled from sources throughout Colorado to test for the relative importance of various predictors of sport fish Hg concentrations related to the factors described above. We used available system-specific predictors as indicators of urbanization, food web structure, productivity, and Hg methylation processes. Further, we used our observations combined with those of others to compare rates of sport fish Hg change that might be expected in response to a variety of factors primarily focusing on empirical changes in food webs and hypothetical changes in Hg emissions. Our comparisons provide a basis to inform decisions about the magnitude and relevant spatial and temporal scales for developing appropriate fish consumption advice.

## Materials and methods

### Data collection and compilation

**Fig 1:**
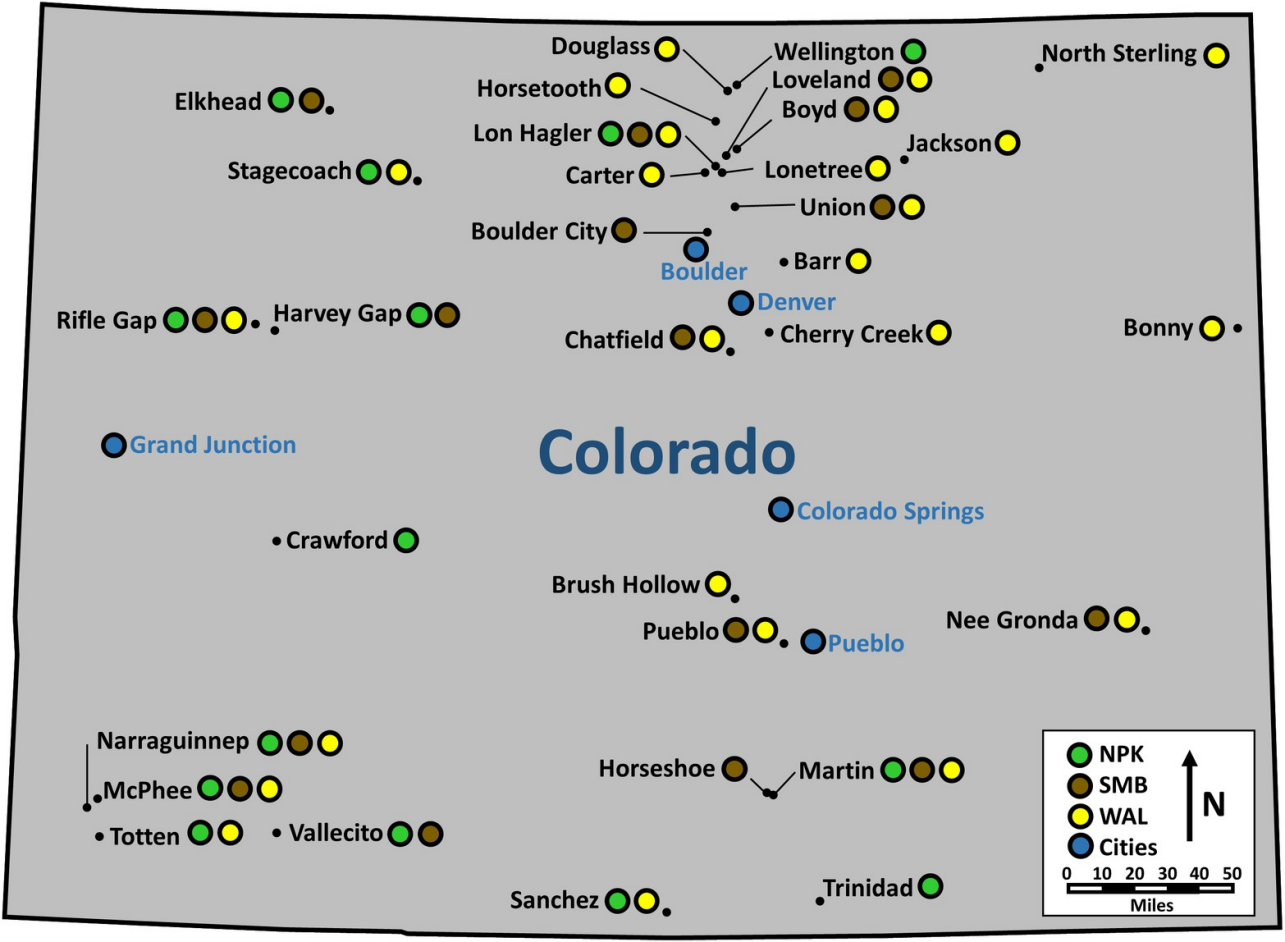
Map of Colorado reservoirs and sport fish species used for analyses. Reservoirs are labelled and shown as small black circles. Larger colored circles represent the fish species tested in each reservoir, green represents northern pike, brown represents smallmouth bass and yellow represents walleye.

Sport fish Hg concentrations (2004 to 2013) available from Colorado Department of Public Health and Environment (CDPHE) were used as response variables for three fish species—northern pike (*Esox lucius*), smallmouth bass (*Micropterus dolomieu*), and walleye (*Sander vitreus*)—across 32 reservoirs (Fig 1). For each system-species combination, simple linear regression was used to estimate fish Hg concentrations (and upper 95% confidence intervals when regressions were based on five or more values) at lengths that were related to the most commonly applied length-based harvest regulations (i.e., minimum length limits) in Colorado (864 mm for northern pike and 381 mm for smallmouth bass and walleye). We expected sport fish Hg concentrations to increase, decrease, or remain the same with increasing lengths depending on the characteristics of a given reservoir. Thus, estimates of fish Hg concentrations at their respective, relevant lengths were used whether the regression slope was positive, negative, or not significantly different than zero. To develop the regression analyses and determine Hg at the specified lengths, data from individual fish and composite samples were used. Composite samples were represented by a single datum as the mean length of the group of fish tested and the resulting Hg concentration. Individual fish lengths in composite samples were within 5% of the measured mean for all fish included in any given composite sample. Measurements of Hg concentration that fell below detection limits (variable across years) were censored by assigning a value equal to half the detection limit [41]. Regression analyses for single system-species combinations were never based solely on data that fell below a Hg concentration detection limit of 0.3 ppm because it was felt the assigned value (0.15 ppm) could mask a relatively large amount of variability (potential empirical range from 0 to 0.3). In 26 of 730 cases (less than one half percent of the data available), censored values (0.15 ppm) for the detection limit of 0.3 ppm were applied. In these cases there were additional data available to corroborate/support the censored data. Since censored values were similar to uncensored information, results were not sensitive to the inclusion or removal of these 26 values in regression analyses, or overall findings. When less than five individual or composite samples were available for a given system-species combination, those estimates were only retained if data from other species within the same system, or data from the same system but outside the 2004–2013 timeframe, corroborated the estimate. Samples were collected from different systems in different years and with different methodologies (i.e., sample sizes, composites versus individuals, species analyzed) and these are described in Table 1.

**Table 1:** Hg sample descriptions by system and species.

Reservoir	NPK	SMB	WAL	Year(s)	Individuals	Composites	Censored		Just write in extrapolation by species, there aren’t many					
CRAWFORD RESERVOIR	5			2008	1N	4N (2–3)								
ELKHEAD RESERVOIR	8			2005, 2007	1N	7N (4–5)								
HARVEY GAP RESERVOIR	9			2007, 2013	6N	3N (2)	2N (0.1)							
LON HAGLER RESERVOIR	2			2007, 2009	2N		1N (0.1)							
MARTIN LAKE	9			2005	9N		8N (0.01)							
McPHEE RESERVOIR	1			2010	1N									
NARRAGUINNEP RESERVOIR	4			2005	4N									
RIFLE GAP RESERVOIR	22			2007, 2008	4N	18N (2–5)	1N (0.1)							
SANCHEZ RESERVOIR	2			2004	2N									
STAGECOACH RESERVOIR	3			2007		3N (2–3)								
TOTTEN RESERVOIR	19			2005	19N		15N (0.1)							
VALLECITO RESERVOIR	23			2004, 2012	8N	15N (2)	11N (0.3)							
WELLINGTON RESERVOIR	1			2007	1N		1N (0.1)		Exprapolation in text.					
**Reservoir**	**NPK**	**SMB**	**WAL**	**Year(s)**	**Individuals**	**Composites**	**Censored**	**Obs (ppb)**	**Total**	**Watercode**	**Reservoir**	**NPK**	**SMB**	**WAL**
**Barr**			9	2004, 2011	9W		1W (0.3)	W (132, 298)	9	53796	**Barr**			x
**Bonny**			11	2005		11W (2–5)	10W (0.1)	W (58, 106)	11	79384	**Bonny**			x
**Boulder City**		2		2007	2S		2S (0.1)	S (50)	2	54089	**Boulder City**		x	
**Boyd**		1	55	2006, 2011, 2012	1S, 55W		14W (0.1), 2W (0.02)	S (110), W (70, 155)	56	52491	**Boyd**		x	x
**Brush Hollow**			15	2004, 2006	5W	10W (6)	1W (0.1)	W (301, 844)	15	79409	**Brush Hollow**			x
**Carter**			68	2006, 2011, 2012	68W			W (299, 425)	68	54255	**Carter**			x
**Chatfield**		12	32	2004, 2013	32W	12S (5)	12S (0.3)	S (150), W (85, 198)	44	54306	**Chatfield**		x	x
**Cherry Creek**			12	2007		12W (4–5)	12W (0.1)	W (50)	12	52580	**Cherry Creek**			x
**Crawford**	5			2008	1N	4N (2–3)		N (198, 330)	5	89284	**Crawford**	x		
**Douglass**			20	2007, 2012	17W	3W (5)	4W (0.02), 3W (0.1)	W (38, 96)	20	58695	**Douglass**			x
**Elkhead**	8	3		2005, 2007	1N	7N (4–5), 3S (3–4)		N (20, 396), S (660, 886)	11	66438	**Elkhead**	x	x	
**Harvey Gap**	9	4		2007, 2013	6N, 2S	3N (2), 2S (2–5)	2N, 1S (0.1)	N (166, 298), S (328, 563)	13	67226	**Harvey Gap**	x	x	
**Horseshoe**		16		2005, 2006	1S	15S (2–3)		S (420, 693)	16	79803	**Horseshoe**		x	
**Horsetooth**			18	2006		18W (3–4)		W (557, 1102)	18	55168	**Horsetooth**			x
**Jackson**			8	2006		8W (4–5)		W (103, 183)	8	53037	**Jackson**			x
**Loveland**		2	3	2008		2S (2), 2W (4–5)	1S, 3W (0.1)	S (105), W (50)	5	53176	**Loveland**		x	x
**Lonetree**			56	2011, 2012	34W	22W (2)	2W (0.02)	W (102, 151)	56	53152	**Lonetree**			x
**Lon Hagler**	2	2	6	2007, 2009, 2013	2N, 2S, 6W		1N (0.1)	N (88), S (241), W (61, 98)	10	55562	**Lon Hagler**	x	x	x
**Martin**	9	3	4	2005	9N, 3S	4W (4)	8N, 1S, 2W (0.01)	N (30, 152), S (291), W (10)	16	79841	**Martin**	x	x	x
**McPhee**	1	41	35	2004, 2010, 2012	1N, 29S, 27W	12S (5), 8W (3–5)		N (440), S (618, 816), W (339, 478)	77	94273	**McPhee**	x	x	x
**Narraguinnep**	4	4	25	2005, 2013	4N, 4S, 25W			N (1290), S (347), W (340, 522)	33	91481	**Narraguinnep**	x	x	x
**Nee Gronda**		3	11	2005	3S, 11W		3S, 11W (0.1)	S (50), W (50)	14	79613	**Nee Gronda**		x	x
**North Sterling**			37	2013	37W		15W (0.02)	W (45, 60)	37	53328	**North Sterling**			x
**Pueblo**		6	10	2013	6S, 10W		1S, 8W (0.02)	S (75, 377), W (13, 43)	16	81783	**Pueblo**		x	x
**Rifle Gap**	22	9	4	2007, 2008	4N, 4S, 2W	18N, 9S (2–5), 2W (4)	1N (0.1)	N (260, 440), S (585, 790), W (351)	35	69422	**Rifle Gap**	x	x	x
**Sanchez**	2		12	2004	2N, 12W		2W (0.03)	N (764), W (124, 1006)	14	92128	**Sanchez**	x		x
**Stagecoach**	3		2	2007		3N (2–3), 2W (3)		N (233), W (125)	5	73902	**Stagecoach**	x		x
**Totten**	19		3	2005	19N, 3W		15N (0.1)	N (120, 233), W (95)	22	92659	**Totten**	x		x
**Trinidad**			18	2004, 2007		18W (2–5)	1W (0.1)	W (280, 432)	18	81911	**Trinidad**			x
**Union**		1	11	2006	1N, 11W		1N, 10W (0.1)	S (50), W (86, 121)	12	53570	**Union**		x	x
**Vallecito**	23		28	2004, 2012	8N, 22W	15N, 6W (2)	11N, 2W (0.3)	N (496, 846), W (343, 819)	51	92902	**Vallecito**	x		x
**Wellington**	1			2007	1N		1N (0.1)	N (50)	1	56906	**Wellington**	x		

Each study reservoir is listed and sample sizes for northern pike (NPK), smallmouth bass (SMB), and walleye (WAL) are provided along with the year or years data were collected. Individuals that were tested for Hg concentration are indicated with a sample size followed by the letter corresponding to the first letter of the species represented. Composite samples are indicated with a sample size followed by the letter corresponding to the first letter of the species represented (N, S, and W for northern pike, smallmouth bass, and walleye, respectively) and a parenthetical range of individuals per composite sample. In systems where empirical data were not available for a given species, that system was excluded from that species analysis. Data that were censored are indicated with a sample size followed by the letter corresponding to first letter of the species represented and a parenthetical detection limit relevant to those data. Estimates of fish Hg concentrations (ppb) from empirical data (Obs) are provided following the letter corresponding to the first letter of the species represented as well as the upper 95% confidence interval (parenthetically, following the Hg concentration) when five or more samples were available to calculate error for a given system-species combination. Where error could not be calculated, only the estimated Hg concentration (ppb) is provided parenthetically without the upper 95% confidence interval.

We selected a regression approach to maximize the amount of data that could be included in these analyses and to represent a length that is informative to the health of anglers and their families that harvest sport fish in Colorado. Our approach generally limited extrapolation outside Hg concentrations estimated for 864 mm northern pike and 381 mm smallmouth bass and walleye. However, extrapolation (using data from fish that did not encompass the length where point estimates were made) was necessary for some (described below) of the 54 reservoir-species combinations. These combinations, (and the range of fish lengths [or individual fish length] that did not encompass the point estimate) for northern pike (864 mm) included Lon Hagler (475–591 mm), McPhee (480 mm), Narraguinnep (400–840 mm), Totten (428–822 mm), and Wellington (490 mm). For smallmouth bass (381 mm) these combinations included Boulder City (243 mm), Boyd (280 mm), Chatfield (165–300 mm), Harvey Gap (163–351 mm), Lon Hagler (209–308 mm), Martin (242–285 mm), Nee Gronda (182–275 mm), Pueblo (280–360), Rifle Gap (122–338 mm), and Union (245 mm). For walleye (381 mm) these combinations included Rifle Gap (461–525 mm), Stagecoach (388-457mm), and Totten (520–635 mm). These system-species combinations were retained because additional data (after 2013 or from other fish species) corroborated point estimates, and including these system-species combinations allowed us to produce predictions there. We note that this was done for prediction purposes to inform fish Hg testing, and would not be appropriate to develop fish consumption advisories to protect human health.

We used data from 32 disparate reservoirs across Colorado with a variety of municipal, agricultural, and recreational uses. Predictors of the response variables (fish Hg concentrations at the specified lengths) were selected based on previous research, ease of data collection, and data availability. We primarily used data that were accessible to us, and also relatively easily collected or calculated metrics, so others could use analogous measurements across a variety of systems, potentially with data already available. We acknowledge that some of these metrics are oversimplified, but complex community, atmospheric and watershed-level modeling to capture Hg inputs and cycling were outside the scope of this study. Thus, metrics used for predictions should be selected with forethought, and potential confounding factors should be considered. We had *a priori* hypotheses associated with each predictor variable, and for each system, predictors included a group of 17 indices developed from a compilation of data from 2000 to 2014. These covariates included: 1) system surface area (Area; ha), 2) shortest linear distance to Colorado Interstate 25 (I-25; km), 3) population density (people/ha) of the county containing the system (Pop dens), 4) the total mass of salmonids stocked from 2003–2013 (Salm mass; kg), 5) the mean annual stocking density of rainbow trout (*Oncorhynchus mykiss*) from 2003–2013 (RBT kg/ha), 6) whether or not rainbow trout were stocked from 2003–2013 (RBT), 7) presence or absence of bluegill (BGL, *Lepomis macrochirus*), 8) presence or absence of cyprinids (CYP), 9) presence or absence of gizzard shad (GSD, *Dorosoma cepedianum*), 10) presence or absence of green sunfish (SNF, *Lepomis cyanellus*), 11) presence or absence of yellow perch (YPE, *Perca flavescens*), 12) index of water level fluctuation (H_2_O ↑↓) with low (0–1 m), medium (1–5 m), and high (> 5 m) fluctuations designated categorically as 0, 1, and 2, respectively, 13) index of chlorophyll a (Chl a; μg/L), 14) index of Secchi depth (Secc; m), 15) index of total phosphorous (TP; mg/L), 16) system elevation (Elev; m), 17) system latitude (Lat).

Data compiled for these analyses were collected intermittently by different agencies and personnel from 2000–2014 depending on a variety of factors including research or monitoring objectives, resource availability, and sampling conditions. We used these data to develop system-specific indices to characterize covariates and test for their relative importance for predicting empirical sport fish Hg concentrations. When possible, we limited data use from 2003–2013 to develop the most relevant covariates for the 2004–2013 Hg concentration data for Colorado sport fish. We did this to ensure that sport fish Hg concentrations were temporally reflective of the system-specific conditions characterized by the indices/covariates. However, contemporaneous data were not always available, and supplemental data from 2000–2002 and 2014 were necessary to characterize some systems (see Table 2 for covariate descriptions). Thus, our indices/covariates were developed to inform the relative importance of past drivers of sport fish Hg concentrations, and design future monitoring efforts, but not to develop fish consumption advice directly.

**Table 2:** Predictive variables of sport fish Hg, the mechanism they operate under and their hypothesized influence on fish Hg concentrations.

Predictor	Mechanism	Expectation	Data source	Time frame
**Area**	Increased depositional area	+	CPW	Contemporary
**I-25**	Increased loading/urbanization	-	Google Earth	Contemporary
**Pop dens**	Increased loading/urbanization	+	2010 census	2010
**Salm mass**	Stocked forage/biomass dilution	-	CPW	2003–2013
**RBT kg/ha**	Stocked forage/biomass dilution	-	CPW	2003–2013
**RBT**	Stocked forage/biomass dilution	-	CPW	2003–2013
**BGL**	Forage base/biomass dilution	-	CPW	2003–2013
**CYP**	Forage base/biomass dilution	-	CPW	2003–2013
**GSD**	Forage base/biomass dilution	-	CPW	2003–2013
**SNF**	Forage base/biomass dilution	-	CPW	2003–2013
**YPE**	Forage base/biomass dilution	-	CPW	2003–2013
**H**_**2**_ **O ↑↓**	Methylation/bioavailability	+	CPW	Contemporary
**Chl a**	Nutrients/biomass dilution	-	CDPHE	2000–2013
**Secc**	Nutrients/biomass dilution	+	CDPHE	2000–2013
**TP**	Nutrients/biomass dilution	-	CDPHE	2000–2013
**Elev**	Growing season/biomass dilution	+	CPW	Contemporary
**Lat**	Growing season/biomass dilution	+	CPW	Contemporary

Development of these covariates is described in detail in section *2*.*1*. *Data collection and compilation*. Abbreviations for the various predictive covariates are: System surface area (Area; ha), shortest linear distance to Colorado Interstate 25 (I-25; km), population density (people per hectare) of the county containing the system (Pop dens), the total mass of salmonids stocked from 2003–2013 (Salm mass; kg), the mean annual stocking density (kg/ha) of rainbow trout (*Oncorhynchus mykiss*) from 2003–2013 (RBT kg/ha), whether or not rainbow trout were stocked from 2003–2013 (RBT), presence or absence of bluegill (BGL, *Lepomis macrochirus*), presence or absence of cyprinids (CYP), presence or absence of gizzard shad (GSD, *Dorosoma cepedianum*), presence or absence of green sunfish (SNF, *Lepomis cyanellus*), presence or absence of yellow perch (YPE, *Perca flavescens*), index of water level fluctuation (H_2_O ↑↓) with low (0–1 m), medium (1–5 m), and high (> 5 m) fluctuation designated categorically as 0, 1, and 2, respectively, index of chlorophyll a (Chl a; μg/L), index of Secchi depth (Secc; m), index of total phosphorous (TP; mg/L), system elevation (Elev; m), and system latitude (Lat). Mechanism, directional expectation of fish Hg concentrations, and data sources and their respective time frames are provided (CPW: Colorado Parks and Wildlife, CDPHE: Colorado Department of Public Health and Environment).

Data from multiple system-specific surveys and sampling events were available from CPW from 2003–2013 to inform a subset of covariates including, Area, I-25, Salm mass, RBT kg/ha, RBT, BGL, CYP, GSD, SNF, YPE, H_2_O ↑↓, Elev, and Lat. Forage fish species were assessed as present (captured during any CPW survey from 2003–2013), or absent (not captured during any CPW survey from 2003–2013). The qualitative water level fluctuation index (H_2_O ↑↓) was based on information provided by CPW biologists from 2003–2013. Water quality data (Chl a, Secc, and TP) were collected by multiple agencies including the United States Geological Survey, the Bureau of Reclamation, and CDPHE. These data were made available by CDPHE (R. Anthony and K. Richardson, pers. comm.). Data collected during summer (May-September inclusive) 2000–2014 were used to calculate mean, system-specific indices to characterize these water quality covariates. Nutrient data (Chl a, and TP concentrations) below detection limits were censored by assigning a value equal to half the detection limit [41]. Values/categorizations of these metrics are provided for clarity and comparison (Table 3). Data were acquired from public sources, and fish Hg concentration data are available for download from CDPHE (https://cdphe.colorado.gov/water-quality-records-center-and-requests). The predictors of fish Hg concentrations tested for each reservoir are provided in Table 3.

**Table 3:** Reservoir metrics and characteristics.

Reservoir	Area	I-25	Pop dens	Salm mass	RBT kg/ha	Forage	H_2_O	Chl a	Secc	TP	Elev	Lat
**Barr**	0	17	0	0	1	RBT, CYP, GSD, YLP	2	62.90	1.75	0.59	0	4421839
**Bonny**	0	231	0	0	0	CYP, GSD, YLP	0	41.60	0.25	0.13	0	4388661
**Boulder City**	0	20	0	0	0	RBT, BGL, CYP, GSD, SNF, YLP	1	3.81	1.27	0.01	0	4436495
**Boyd**	0	3	0	0	1	RBT, BGL, CYP, GSD, YLP	2	4.61	2.16	0.01	0	4475682
**Brush Hollow**	0	38	0	0	9	RBT, BGL, CYP, GSD, SNF, YLP	2	12.20	1.79	0.03	0	4257259
**Carter**	0	19	0	0	1	RBT, BGL, YLP	2	1.68	2.85	0.01	0	4464785
**Chatfield**	0	15	0	0	1	RBT, BGL, CYP, GSD, YLP	2	7.28	2.23	0.02	0	4377856
**Cherry Creek**	0	3	0	0	2	RBT, BGL, CYP, GSD, YLP	2	16.21	1.02	0.09	0	4387901
**Crawford**	0	251	0	0	105	RBT, CYP, YLP	2	16.33	3.10	0.02	0	4285036
**Douglas**	0	6	0	0	2	RBT, BGL, CYP, GSD, SNF, YLP	1	6.08	0.95	0.01	0	4505667
**Elkhead**	0	198	0	0	90	RBT, CYP, BLG, YLP	1	1.89	1.39	0.02	0	4493826
**Harvey Gap**	0	239	0	0	435	RBT, BLG, YLP	2	36.50	1.42	0.16	0	4388177
**Horseshoe**	0	8	0	0	943	RBT, BGL, CYP, GSD, YLP	2	7.49	1.11	0.02	0	4162141
**Horsetooth**	0	13	0	0	0	RBT, BGL, CYP, GSD, YLP	2	3.14	2.23	0.01	0	4489770
**Jackson**	0	77	0	0	1	RBT, BGL, CYP, GSD, YLP	2	22.17	0.70	0.14	0	4471765
**Loveland**	0	7	0	0	1	RBT, BGL, CYP, GSD, SNF, YLP	2	4.91	2.80	0.02	0	4473361
**Lonetree**	0	12	0	0	0	BGL, CYP, GSD, YLP	2	4.50	2.31	0.02	0	4465199
**Lon Hagler**	0	13	0	0	8	RBT, BGL, CYP, GSD, SNF, YLP	2	5.47	1.64	0.01	0	4468354
**Martin**	0	7	0	0	8	RBT, BGL, CYP, GSD, YLP	0	3.63	1.50	0.03	0	4162550
**McPhee**	0	337	0	0	0	RBT, CYP, YLP	2	2.21	3.27	0.00	0	4157577
**Narraguinnep**	0	353	0	0	0	YLP	1	1.05	2.14	0.00	0	4155345
**Nee Gronda**	0	161	0	0	0	BGL, CYP, GSD, YLP	0	8.82	1.06	0.02	0	4241616
**North Sterling**	0	140	0	0	1	RBT, BGL, CYP, GSD, YLP	2	53.14	0.85	0.15	0	4514409
**Pueblo**	0	11	0	0	0	RBT, BGL, CYP, GSD, YLP	2	4.53	3.06	0.01	0	4235598
**Rifle Gap**	0	225	0	0	7	RBT, BGL, CYP, YLP	2	1.14	3.55	0.02	0	4390337
**Sanchez**	0	79	0	0	0	CYP, SNF	2	3.40	1.50	0.07	0	4104313
**Stagecoach**	0	158	0	0	5	RBT	1	6.13	2.98	0.06	0	4460391
**Totten**	0	354	0	0	5	RBT, BGL, SNF, YLP	1	1.28	3.00	0.00	0	4141204
**Trinidad**	0	3	0	0	2	RBT, BGL, GSD, YLP	1	2.32	1.57	0.01	0	4110224
**Union**	0	4	0	0	2	RBT, BGL, CYP, GSD, SNF, YLP	1	3.96	1.24	0.02	0	4447762
**Vallecito**	0	260	0	0	1	RBT, YLP	1	1.46	4.16	0.01	0	4141594
**Wellington**	0	3	0	0	13	RBT, BGL, CYP, GSD, YLP	2	1.08	1.75	0.01	0	4507498

Abbreviations for the various covariates are provided in Table 2. Prey species are grouped and listed under the Forage category represented by their corresponding three letter code.

### Data analyses

We applied the machine learning technique called random forest (RF) to statistically test for indices/covariates as predictors of sport fish Hg concentrations within the dataset and to test for the relative importance and effectiveness of the predictors for characterizing sport fish Hg concentrations [42]. The RF approach is ideal because it is based on a form of cross-validation, is entirely nonlinear, accounts for obscure interactions, and simultaneously evaluates multicollinear variables [42,43]. Although many other approaches prevent the inclusion of multicollinear variables, the RF approach can incorporate all readily available data for prediction. For example, TP and Chl a are often highly correlated, and with most approaches, one variable or the other would need to be excluded from any single predictive model. Using the RF approach allowed us to incorporate all variables of interest despite potential multicollinearity.

The RF approach falls under a much larger class of machine learning methods where prediction is of primary interest. These methods rely on “bagging,” which refers to the method of fitting regression trees to numerous bootstrapped versions of the training data (i.e., observations sampled with replacement from the larger data set). The resulting trees are then averaged to yield a predictor with low bias and variance. To perform our RF analyses, we used the ‘randomForest’ package 4.6–6 [44] in R [45]. In the RF implementation described here, 2,000 regression trees were used to calculate accuracies and error rates for each observation using out-of-bag predictions (i.e., predicting data that were withheld from each tree). The predictions of data that were not used to evaluate fit can be considered as a form of cross-validation. Variable importance can then be assessed by comparing the increase in; 1) mean squared prediction error, and 2) node purity associated with each individual covariate. We refer the interested reader to several works for more information on these variable importance metrics [44–46].

For each fish species, a full model (all predictors) was used to test for predictive power and variable importance. Although we selected metrics that were relatively common and available, we were aware that some of the predictors used may not be available. To determine how well this approach performed when only the best predictors were used, we removed most predictors from the full models and repeated the RF statistical analyses on a condensed model for each species. Predictors were retained if they were found to be important as measured by the increase in mean square prediction error and node purity. These selections were made somewhat arbitrarily for demonstration purposes, however, *a priori* hypotheses, and support from other research were considered along with the predictor importance metrics when full models were condensed to the most important three or four variables. For each system-species combination, we compared the estimated Hg concentrations from empirical data to the Hg concentrations estimated from the full RF model. The difference of these (empirical estimate minus RF prediction) was regressed as a function of the empirically estimated Hg concentrations to test whether this differed from a one-to-one relationship to evaluate the presence of any bias. In general, we expected that the best predictors of Hg concentrations in 864 mm northern pike would be those related to stocked salmonids as forage while the best predictors of Hg concentrations in 381 mm smallmouth bass and walleye would be more dependent on system productivity metrics.

### Prediction application

The food web structure in Elkhead Reservoir (Colorado, USA) changed significantly when the stocking of salmonids (up to 3,400 kg of ~ 250 mm rainbow trout stocked in a single year; 2010, with a mean of about 1,500 kg of salmonids stocked each year) was discontinued in the fall of 2011. Based on previous whole-system experimental work [20] we expected northern pike Hg concentrations in this reservoir to increase from this management action. For comparison, northern pike Hg concentrations were available from 2005 and 2007, prior to this management shift when significant masses of catchable (~225 mm) rainbow trout were being stocked. We used the full and condensed models developed for northern pike to predict what 864 mm northern pike Hg concentrations would be under these new conditions (setting all salmonid stocking to zero). The output provided a value for comparison to empirical northern pike Hg concentration data that were collected late in 2013 (following the cessation of stocking), that was not included as training data for the RF approach. This provided information from the same system with and without salmonid stocking (a factor expected to have predictive importance for large northern pike) to evaluate model performance, recognizing that the food web structure may or may not have been in equilibrium following this shift.

## Results

### Random forest modeling

**Fig 2:**
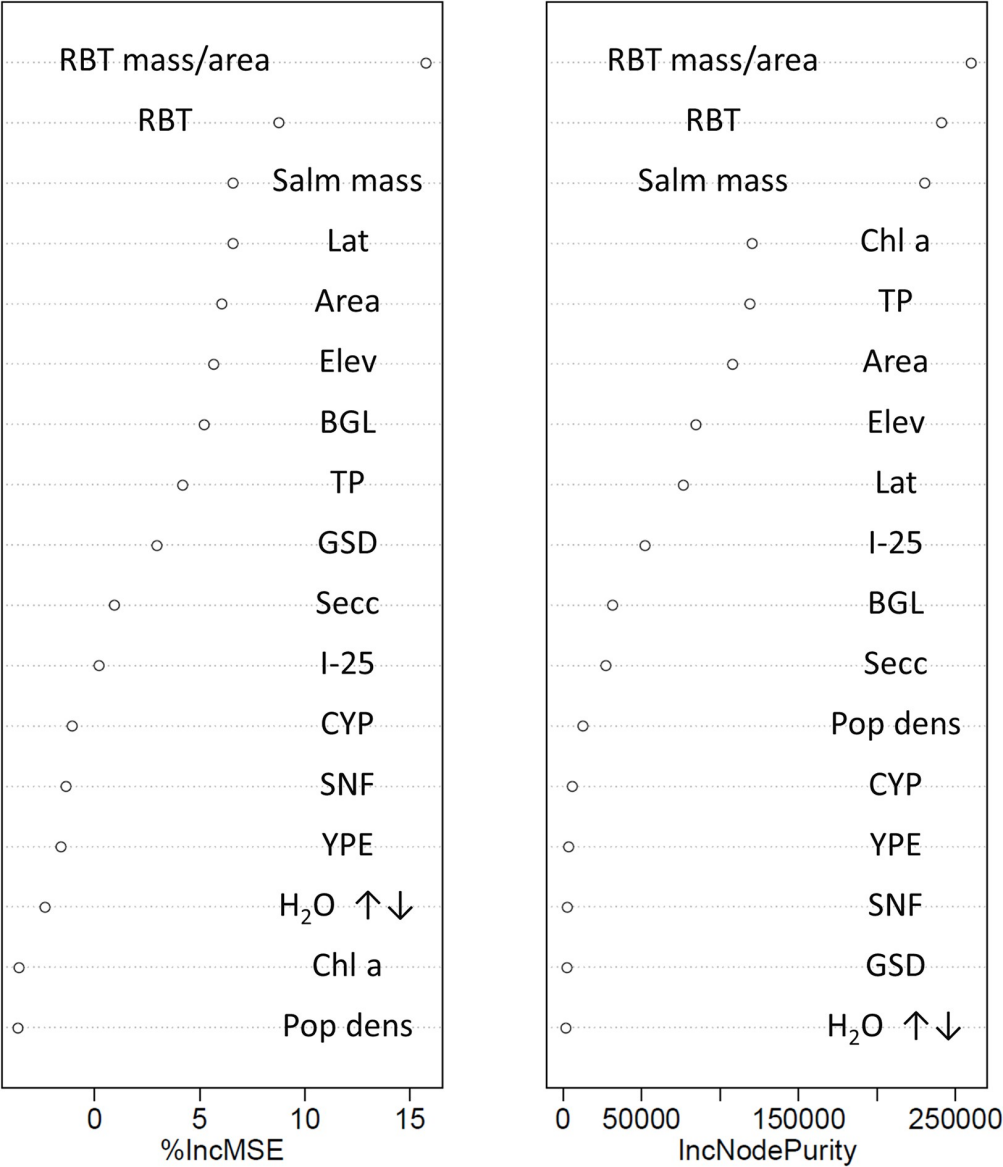
Random forest variable importance indicators for the full model (all variables) predicting 864 mm northern pike Hg concentrations from thirteen Colorado Reservoirs. Variables are listed top to bottom in order of relative predictive value from most to least, respectively. Abbreviations for the various predictive covariates are provided in Table 2.

**Fig 3:**
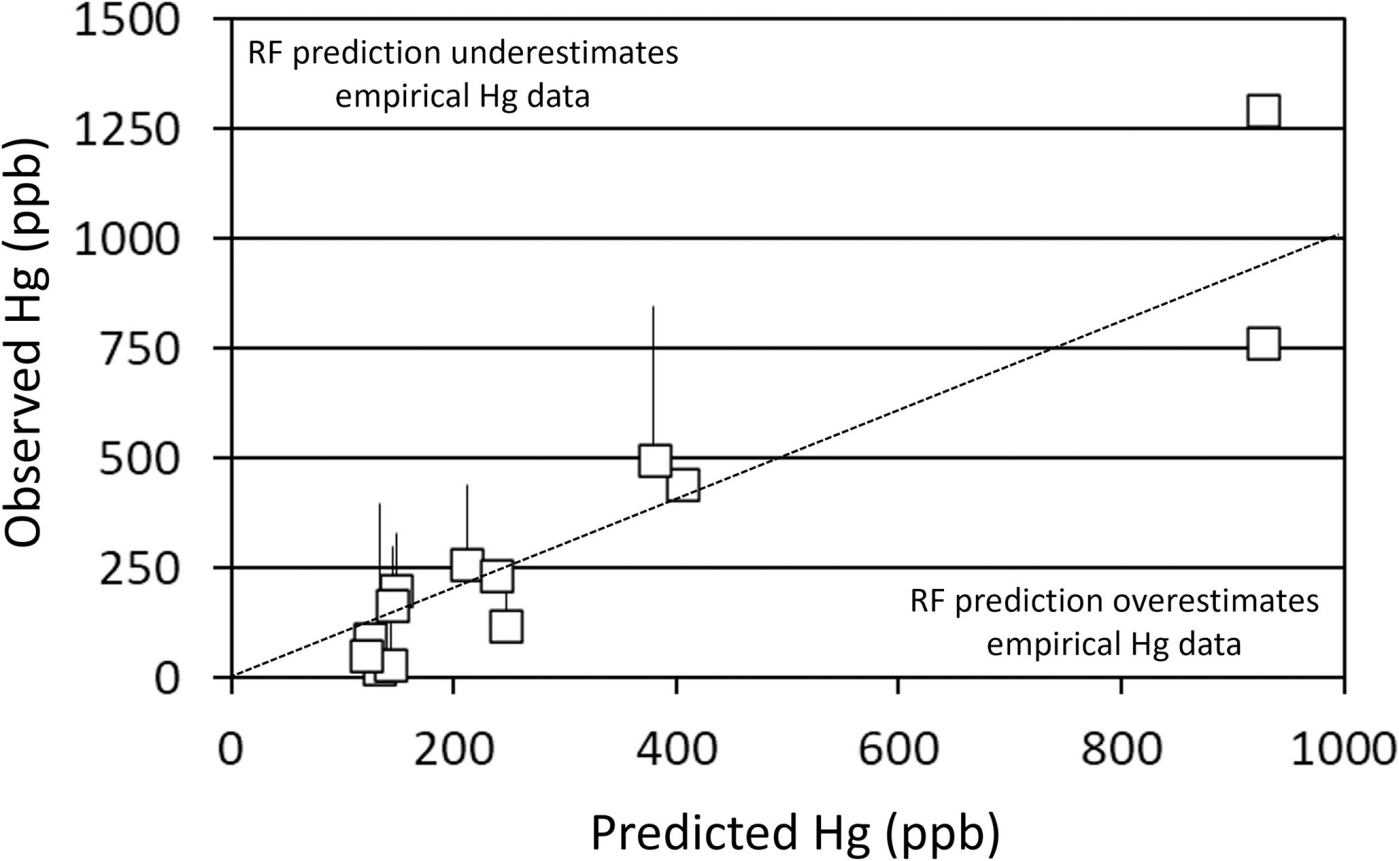
Predicted Hg concentrations of 864 mm northern pike (*Esox lucius*). Estimated northern pike Hg concentrations from empirical data from thirteen Colorado reservoirs are plotted as a function of predicted Hg concentrations from the Random Forest (RF) approach. Regions of the plot where RF predictions overestimate and underestimate estimates from empirical data are indicated. Upper 95% confidence intervals are provided for systems with empirical estimates based on five or more samples. The condensed model (the three salmonid stocking metrics only) was used in this analysis. A one-to-one (dotted) line is provided for comparison.

Salmonid stocking metrics best explained 864 mm northern pike Hg concentrations. Thirteen systems were included in the 864 mm northern pike RF analysis. The Hg concentration in northern pike was also predicted from Elkhead Reservoir experiencing no salmonid stocking. The full model explained about 25% of the variability in northern pike Hg concentrations with all three salmonid stocking metrics being the most important predictors of northern pike Hg concentrations (Fig 2). To reduce the number of predictive variables in the model, only the top three predictors related to stocking were included in a second model. This more parsimonious, condensed model (northern pike Hg concentration as a function of Salm mass, RBT kg/ha, and RBT) explained about 50% of the variability in the data. There was a consistent bias from a one-to-one line (slope; 0.33, F = 28.79, p-value < 0.01) where predicted values were higher than those observed at relatively low concentrations and lower than those observed at relatively high concentrations (Fig 3).

**Fig 4:**
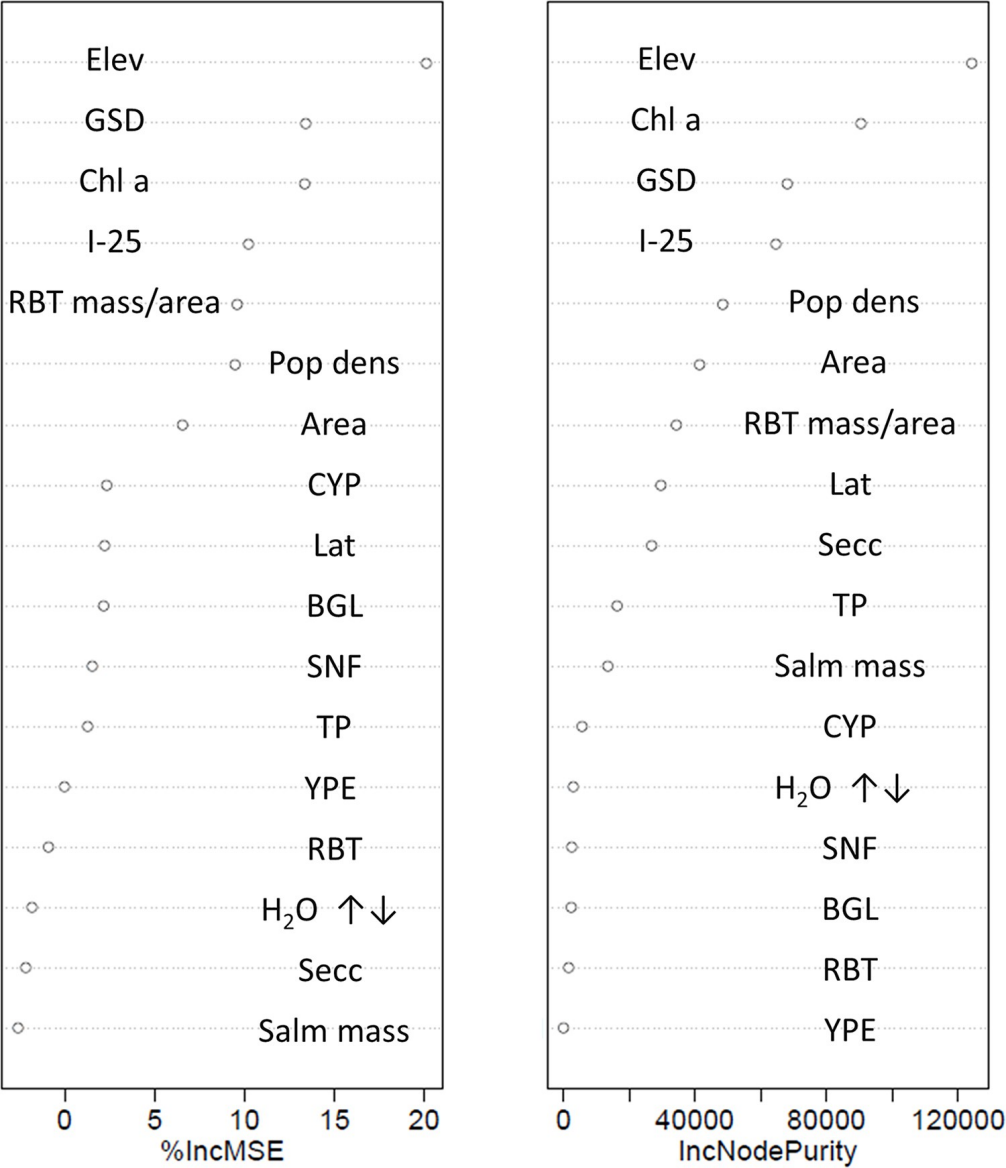
Random forest variable importance indicators for the full model (all variables) predicting 381 mm smallmouth bass (*Micropterus dolomieu*) Hg concentrations from fifteen Colorado Reservoirs. Variables are listed top to bottom in order of relative predictive value from most to least, respectively. Abbreviations for the various predictive covariates are provided in Table 2.

**Fig 5:**
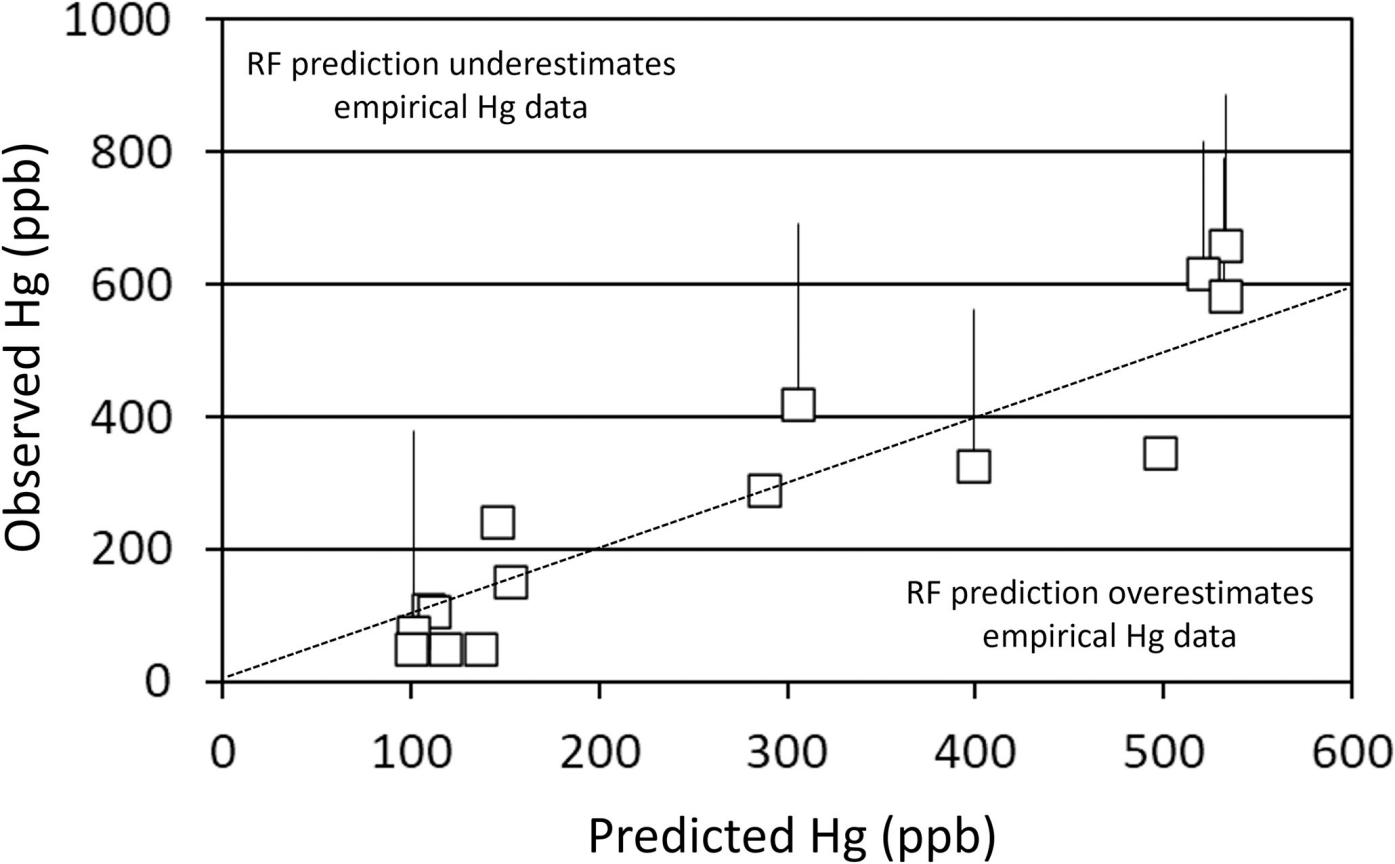
Predicted Hg concentrations of 381 mm smallmouth bass (*Micropterus dolomieu*). Estimated smallmouth bass Hg concentrations from empirical data from fifteen Colorado reservoirs are plotted as a function of predicted Hg concentrations from the Random Forest (RF) approach. Regions of the plot where RF predictions overestimate and underestimate estimates from empirical data are indicated. Upper 95% confidence intervals are provided for systems with empirical estimates based on five or more samples. The condensed RF model (Elev, GSD, and Chl a) was used in this analysis. A one-to-one (dotted) line is provided for comparison.

The best predictors of smallmouth bass Hg concentrations were metrics related to growing season, productivity, and forage base. Fifteen systems were included in the smallmouth bass RF analysis. The full model explained about 55% of the variability in smallmouth bass Hg concentrations (Fig 4). Although I-25 appeared as the fourth most important predictor in both variable importance indicators, the relationship was contrary to expectations; smallmouth bass Hg concentrations went up with increasing distance from Colorado Interstate 25. Thus, this predictor was removed from further analyses and the top three predictors (Elev, GSD, and Chl a) were used in the condensed model. The more parsimonious, condensed model (smallmouth bass Hg concentration as a function of Elev, GSD, and Chl a) explained about 70% of the variability in the data. There was a consistent bias from a one-to-one line (slope; 0.25, F = 17.78, p-value < 0.01) where predicted values were higher than those observed at relatively low concentrations and lower than those observed at relatively high concentrations (Fig 5).

**Fig 6:**
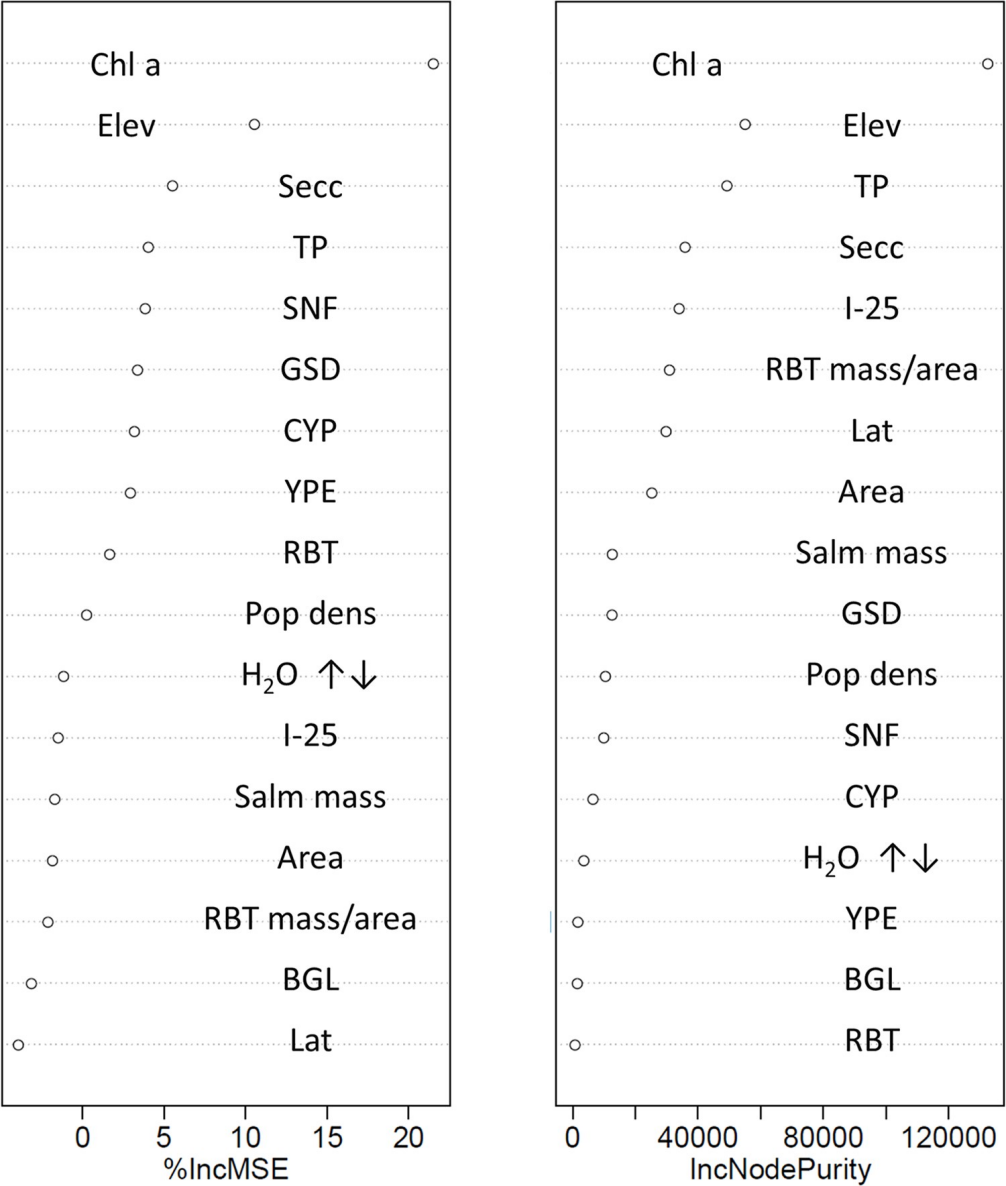
Random forest variable importance indicators for the full model (all variables) predicting 381 mm walleye (*Sander vitreus*) Hg concentrations from 26 Colorado Reservoirs. Variables are listed top to bottom in order of relative predictive value from most to least, respectively. Abbreviations for the various predictive covariates are provided in Table 2.

**Fig 7:**
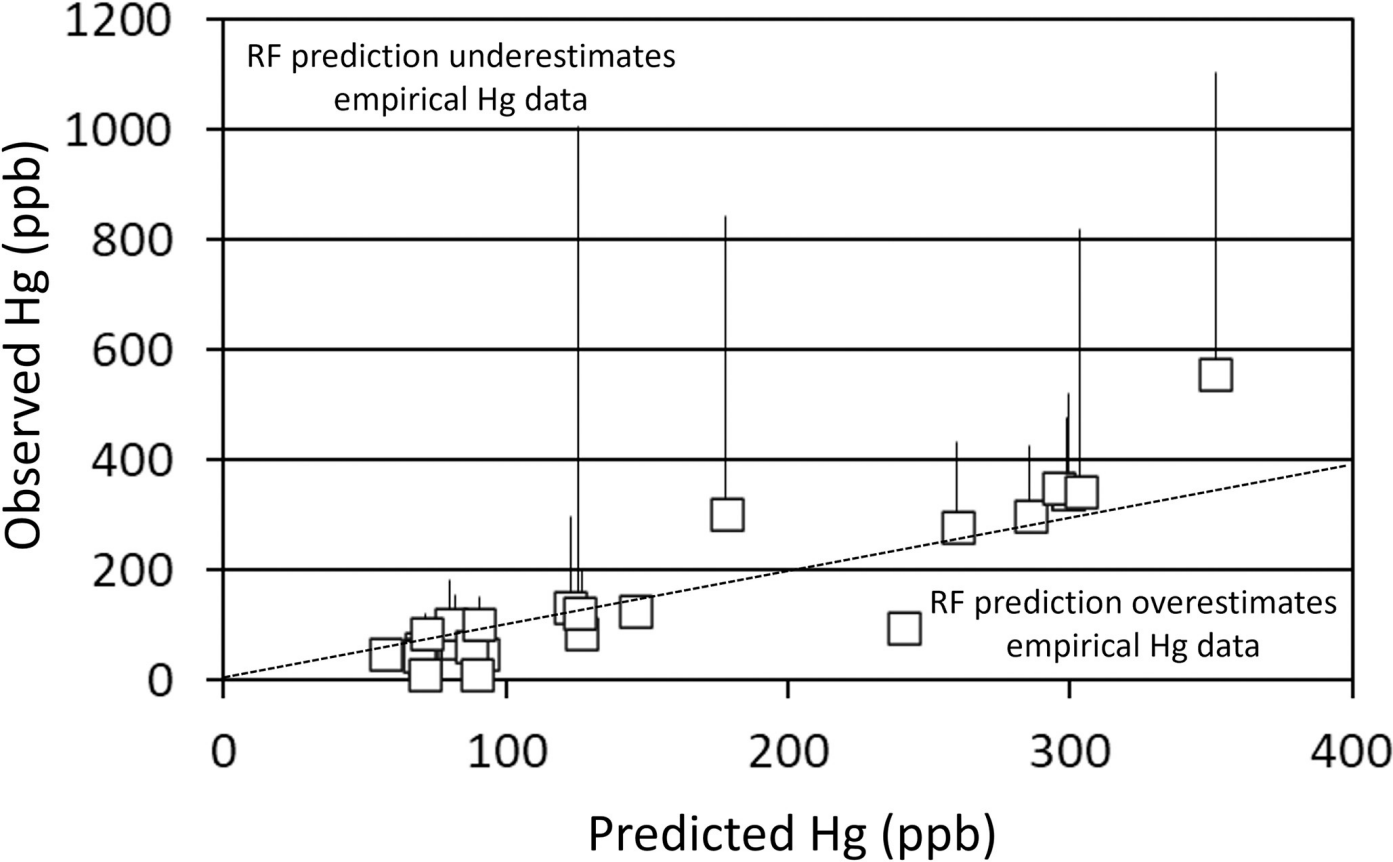
Predicted Hg concentrations of 381 mm walleye (*Sander vitreus*). Estimated walleye Hg concentrations from empirical data from 26 Colorado reservoirs are plotted as a function of predicted Hg concentrations from the Random Forest (RF) approach. Regions of the plot where RF predictions overestimate and underestimate estimates from empirical data are indicated. Upper 95% confidence intervals are provided for systems with empirical estimates based on five or more samples. The condensed model (Chl a, Elev, Secc, and TP) was used in this analysis. A one-to-one (dotted) line is provided for comparison.

Walleye Hg concentrations were best predicted by metrics of system productivity and growing season. Twenty-six systems were included in the walleye RF analysis. The full model explained about 10% of the variability in walleye Hg concentrations (Fig 6). Thus, the top four predictors (Chl a, Elev, Secc, and TP) were used in the condensed model. The more parsimonious, condensed model (walleye Hg concentration as a function of Chl a, Elev, Secc, and TP) explained about 17.5% of the variability in walleye Hg concentrations. There was a consistent bias from a one-to-one line (slope; 0.35, F = 54.17, p-value < 0.01) where predicted values were higher than those observed at relatively low concentrations and lower than those observed at relatively high concentrations (Fig 7).

### Prediction application

The linear regression-based empirical Hg concentration of 864 mm northern pike in Elkhead Reservoir based on 2005–2007 data (during salmonid stocking) was 0.02 ppm. When the full model was applied to predict Hg concentration of 864 mm northern pike in Elkhead Reservoir without stocking (setting Salm mass, RBT mass/area, and RBT to zero in the model), the result was 0.55 ppm. The result from the condensed model (including RBT, Salm mass, and RBT mass/area as predictors) when the same was done (stocking set to zero) was 0.93 ppm. The observed 2013 (salmonid stocking ceased) Hg concentration of 864 mm northern pike in Elkhead Reservoir was 0.57 ppm.

## Discussion

### Random forest modeling

Our expectations based on previous work in Colorado reservoirs (20,21,47,48) regarding the factors important for predicting sport fish Hg concentrations were supported by the RF analysis. The most important predictors (based on the RF variable importance indicators) of 864 mm northern pike Hg concentrations in Colorado were related to salmonid stocking, presumably because of biomass dilution from the consumption of stocked fish that are relatively high in energy and low in Hg content [20,47,48]. We expected that the most important predictors of 381 mm smallmouth bass and walleye Hg concentrations would be related to biomass dilution as well, but driven by system productivity rather than fish stocking. This is likely because 381 mm sport fish are not generally capable of consuming large (> 200 mm) stocked salmonids [49] that represent the majority of the biomass of fish stocked in Colorado. Thus, indicators related to system productivity and growing season (i.e., Elev, Chl a, Secc, TP) were found to be relatively important predictors of smallmouth bass and walleye Hg concentrations compared to stocking indices.

Four variables that were included as predictors of sport fish Hg concentrations were found to be relatively unimportant in the RF analysis. The indicators of proximity to emission sources and deposition potential (Area, I-25, Pop dens) were relatively unimportant as was the indicator of water level fluctuation hypothesized to be positively correlated to Hg methylation [26,27]. Instead, we found that productivity (internal and external sources) in the form of nutrients and forage were better predictors of Hg concentrations in sport fish in this set of systems relative to indicators of Hg deposition and methylation within our dataset. Although studies have shown that Hg sources and deposition may be elevated in and around urban areas, that does not necessarily translate to elevated Hg concentrations in fish within those areas [15,50]. These observations (fish Hg concentrations decoupled from deposition/sources to some degree) could explain some of our findings related to urbanized areas in Colorado.

In Colorado, system elevation is related to several other factors that could potentially increase and decrease fish Hg concentrations. As elevation increases agricultural land-uses decline along with nutrient inputs, precipitation (linked to Hg deposition) increases, growing season declines, and in-lake forage species (e.g., gizzard shad with relatively high energy and low Hg content) are less abundant, which could all lead to elevated sport fish Hg concentrations. However, with increasing elevation, food sources tend to have relatively low trophic positions, food chains tend to be relatively short, and there are generally fewer local sources of Hg emissions, which could all lead to lower sport fish Hg concentrations. In this case, factors related to elevation that led to increased fish Hg concentrations appeared to have a stronger influence than those leading to decreased fish Hg concentrations. The precise mechanism or combination of mechanisms causing this pattern are unknown, but the other three most important predictors of fish Hg concentrations were indicators of system productivity.

An RF approach analogous to what is described here is appropriate to inform and streamline monitoring programs, but not to obtain estimated Hg concentrations as an alternative to direct testing. This technique could be used to identify areas where sport fish Hg concentration shifts may be occurring. Appropriate systems and species could then be prioritized for Hg testing so consumption advisories could be updated accordingly to maximize the benefits of fish consumption and better protect anglers and their families from potential health risks. For example, in response to the management actions and subsequent food web shifts observed in Elkhead Reservoir, testing was prioritized and CDPHE has adjusted fish consumption advice and monitoring efforts there to be more protective of human health.

### Prediction application

The performance of the full and condensed RF models was encouraging. Although there was a range of variability described by the full and condensed models, model results were reasonable despite low sample size, and bias appeared systematic (predicted values were consistently higher than those observed at relatively low concentrations and lower than those observed at relatively high concentrations). The application of the full model to Elkhead Reservoir to predict northern pike Hg concentrations following 2011 (when salmonid stocking was discontinued), resulted in a value within 5% of empirical observations from 2013, while the application of the condensed (three stocking variables) model overestimated northern pike Hg concentrations. Thus, the full model set performed well for making predictions in Elkhead Reservoir specifically, but the condensed model set was more parsimonious and explained more variation in the data across all of the reservoirs considered during the northern pike analysis. It is likely that within Elkhead Reservoir, other predictors included in the full model (in addition to salmonid stocking), may be important for predicting northern pike Hg concentrations, though further investigation at the system-specific level would be necessary to identify mechanism(s). This finding highlights the potential value of the RF approach and including a variety of factors during predictive analyses.

## Conclusions

Many reservoirs in western North America are heavily managed, highly fluctuating, and serve multiple uses which can influence their Hg dynamics [22]. Biota in reservoirs in particular have been found to have elevated Hg concentrations [23–25]. These types of heavily managed and dynamic systems are analogous to those considered here, and Colorado offered a wide range of predictors like high and low elevation and little or no salmonid stocking to heavy salmonid stocking. Thus, the patterns observed in this study may be present in other regions where comparable lake and reservoir management approaches (e.g., salmonid stocking, irrigation, recreation) are being applied. The RF approach described here could identify important predictors of fish Hg concentrations at other locations and scales across the landscape to inform monitoring and advisory programs in place to protect human health.

Based on our observations, sport fish Hg concentrations varied widely across the landscape, driven by multiple, species-specific factors. Thus, simplistic yet flexible approaches to rapidly identify areas or species of concern, and communicating findings to anglers and their families (e.g., the case of the Elkhead Reservoir northern pike population), are likely the most effective means for addressing human health concerns from Hg contamination in the short-term. Based on available atmospheric information [14,51,52], Hg deposition may increase in some areas (including areas of Colorado) in the future. These potential changes coupled with the observed variation in sport fish Hg concentrations, make the RF approach a useful, predictive tool to prioritize and streamline Hg monitoring efforts. Control of anthropomorphic Hg emissions represents an important component of the ubiquitous issue of Hg contamination in the environment. This is recognized by groups like the Minamata Convention on Mercury, and likely represents one of the few long-term solutions to the problem of Hg significantly exceeding natural concentrations in ecosystems and biota. In the short-term, predictive tools like RF that incorporate food web structures, and their dynamics, are important to understand and provide context for Hg bioaccumulation in sport fish and changes in Hg emission, deposition, and cycling.

## Supporting information

S1 File (DOCX)

## References

[1] Driscoll CT, Han Y, Chen CY, Evers DC, Lambert KF, Holsen TM, et al. Mercury contamination in forest and freshwater ecosystems in the Northeastern United States. Bioscience. 2007;57: 17–28.

[2] Mergler D, Anderson HA, Chan LHM, Mahaffey KR, Murray M, Sakamoto M, et al. Methylmercury exposure and health effects in humans: a worldwide concern. Ambio. 2007;36: 3–11. doi: 10.1579/0044-7447(2007)36[3:meahei]2.0.co;2 17408186

[3] Wright LP, Zhang L, Cheng I, Aherne J, Wentworth GR. Impacts and effects indicators of atmospheric deposition of major pollutants to various ecosystems-a review. Aerosol Air Qual Res. 2018;18: 1953–1992.

[4] Pirrone N, Mason R. editors. Mercury fate and transport in the global atmosphere: emissions, measurements and models. New York: Springer; 2009.

[5] Knuth B, Connelly NA, Sheeshka J, Patterson J. Weighing health benefits and health risk information when consuming sport-caught fish. Risk Anal. 2003;23: 1185–1197. 14641893 10.1111/j.0272-4332.2003.00392.x

[6] Nesheim MC, Yaktine AL. editors. Seafood choices: Balancing benefits and risks. Committee on Nutrient Relationships in Seafood: Selections to Balance Benefits and Risks, Food and Nutrition Board. Institute of Medicine of the National Academies. Washington D.C.: The National Academies Press; 2007.

[7] Niederdeppe J, Connelly NA, Lauber TB, Knuth BA. Effects of a personal narrative in messages designed to promote healthy fish consumption among women of childbearing age. Health Commun. 2019;34: 825–837. doi: 10.1080/10410236.2018.1437526 29482372

[8] Wang F, Outridge PM, Feng X, Meng B, Hiembürger-Boavida LE, Mason RP. How closely do mercury trends in fish and other aquatic wildlife track those in the atmosphere?–implications for evaluating the effectiveness of the Minamata Convention. Sci Tot Environ. 2019;674: 58–70. doi: 10.1016/j.scitotenv.2019.04.101 31003088

[9] Brigham ME, VanderMeulen DD, Eagles-Smith CA, Krabbenhoft DP, Maki RP, DeWild JF. Long-term trends in regional wet mercury deposition and lacustrine mercury concentrations in four lakes in Voyageurs National Park. Appl Sci. 2021;11: 1879. doi: 10.3390/app11041879

[10] Sorensen JA, Glass GE, Schmidt KW, Huber JK, Rapp GRJ. Airborne mercury deposition and watershed characteristics in relation to mercury concentrations in water sediments plankton and fish of eighty Northern Minnesota lakes USA. Env Science Technol. 1990;24: 1716–1727.

[11] Driscoll CT, Yan C, Schofield CL, Munson R, Holsapple J. The mercury cycle and fish in the Adirondack lakes. Environ Sci Technol. 1994;28: A136–A143. 10.1021/es00052a721 22668475

[12] Eagles-Smith CA, Ackerman JT, Willacker JJ, Tate MT, Lutz MA, Fleck J, et al. Spatial and temporal patterns of mercury concentrations in freshwater fishes across the Western US and Canada. Sci Total Environ. 2016;568: 1171–1184. 27102274 10.1016/j.scitotenv.2016.03.229

[13] Harris RC, Rudd JWM, Amyot M, Babiarz CL, Beaty KG, Blanchfield PJ, et al. Whole-ecosystem study shows rapid fish-mercury response to changes in mercury deposition. P Natl Acad Sci. 2007;42: 16586–16591. doi: 10.1073/pnas.0704186104 17901207 PMC2034227

[14] Lin CJ, Shetty SK, Pan L, Pongprueksa P, Jang C, Chu HW. Source attribution for mercury deposition in the contiguous United States: regional difference and seasonal variation. JAPCA J Air Waste MA. 2012;62: 52–63. doi: 10.1080/10473289.2011.622066 22393810

[15] Wentz DA, Brigham ME, Chasar LC, Lutz MA, Krabbenhoft DP. Mercury in the nation’s streams–levels, trends and implications: U.S. Geological Survey Circular 1395. 2014.

[16] Bodaly RA, Rudd JWM, Fudge RJP, Kelly CA. Mercury concentrations in fish related to size of remote Canadian Shield lakes. Can J Fish Aquat Sci. 1993;50: 980–987.

[17] Power M, Klein GM, Guiguer KRRA, Kwan MKH. Mercury accumulation in the fish community of a sub-Arctic lake in relation to trophic position and carbon sources. J Appl Ecol. 2002;39: 819–830.

[18] Johnston TA, Leggett WC, Bodaly RA, Swanson HK. Temporal changes in mercury bioaccumulation by predatory fishes of boreal lakes following the invasion of an exotic forage fish. Environ Toxicol Chem. 2003;22: 2057–2062. doi: 10.1897/02-265 12959531

[19] Eagles-Smith CA, Suchanek TH, Colwell AE, Anderson NL, Moyle PB. Changes in fish diets and food web mercury bioaccumulation induced by an invasive planktivorous fish. Ecol Appl. 2008;18: A213–A226. doi: 10.1890/06-1415.1 19475926

[20] Lepak JM, Kinzli KD, Fetherman ER, Pate WM, Hansen AG, Gardunio EI, et al. Manipulation of growth to reduce sport fish mercury concentrations on a whole-lake scale. Can J Fish Aquat Sci. 2012;69: 122–135.

[21] Johnson BM, Lepak JM, Wolff BA. Effects of prey assemblage on mercury bioaccumulation in a piscivorous sport fish. Sci Total Environ. 2015;506–507: 330–337. 10.1016/j.scitotenv.2014.10.101 25460967

[22] Willacker JJ, Eagles-Smith CA, Lutz MA, Tate MT, Ackerman JT, Lepak JM. The influence of reservoirs and their water management on fish mercury concentrations in Western North America. Sci Tot Environ. 2016;568: 739–748. 10.1016/j.scitotenv.2016.03.050 27039275

[23] Tremblay A, Lucotte M. Accumulation of total mercury and methyl mercury in insect larvae of hydroelectric reservoirs. Can J Fish Aquat Sci. 1997;54: 832–841.

[24] French KJ, Anderson MR, Scruton DA, Ledrew LJ. Fish mercury levels in relation to characteristics of hydroelectric reservoirs in Newfoundland, Canada. Biogeochemistry. 1998;40: 217–233.

[25] Bodaly RA, Fudge RJP. Uptake of mercury by fish in an experimental boreal reservoir. Arch Environ Con Tox. 1999;37: 103–109. 10.1007/s002449900494 10341047

[26] Sorensen JA, Kallemeyn LW, Sydor M. Relationship between mercury accumulation in young-of-the-year yellow perch and water-level fluctuations. Env Sci Technol. 2005;39: 9237–9243. doi: 10.1021/es050471r 16382948

[27] Selch TM, Hoagstrom CW, Weimer EJ, Duehr JP, Chipps SR. Influence of fluctuating water levels on mercury concentrations in adult walleye. B Environ Contam Tox. 2007;79: 36–40. doi: 10.1007/s00128-007-9229-0 17618374

[28] St. Louis VL, Rudd JWM, Kelly CA, Bodaly RA, Paterson MJ, Beaty KG, et al. The rise and fall of mercury methylation in an experimental reservoir. Environ Sci Technol. 2004;38: 1348–1358. doi: 10.1021/es034424f 15046335

[29] Pickhardt PC, Folt CL, Chen CY, Klaue B, Blum JD. Algal blooms reduce the uptake of toxic methylmercury in freshwater food webs. P Natl Acad Sci. 2002;99: 4419–4423. doi: 10.1073/pnas.072531099 11904388 PMC123663

[30] Chen CY, Folt CL. High plankton densities reduce mercury biomagnification. Environ Sci Technol. 2005;39: 115–121. 15667084

[31] Karimi R, Chen CY, Pickhardt PC, Fisher NS, Folt CL. Stoichiometric controls of mercury dilution by growth. P Natl Acad Sci. 2007;104: 7477–7482. doi: 10.1073/pnas.0611261104 17456601 PMC1863492

[32] Cleckner LB, Garrison PJ, Hurley JP, Olson ML, Krabbenhoft DP. Trophic transfer of methyl mercury in the northern Florida Everglades. Biogeochemistry. 1998;40: 347–361.

[33] Kidd KA, Paterson MJ, Hesslein RH, Muir DCG, Hecky RE. Effects of northern pike (*Esox lucius*) additions on pollutant accumulation and food web structure, as determined by delta C-13 and delta N-15, in a eutrophic and an oligotrophic lake. Can J Fish Aquat Sci. 1999;56: 2193–2202.

[34] Essington TE, Houser JN. The effect of whole-lake nutrient enrichment on mercury concentration in age-1 yellow perch. T Am Fish Socy. 2003;132: 57–68.

[35] Compeau GC, Bartha R. Sulfate-reducing bacteria—principal methylators of mercury in anoxic estuarine sediment. Appl Environ Microb. 1985;50: 498–502. doi: 10.1128/aem.50.2.498-502.1985 16346866 PMC238649

[36] Fleming EJ, Mack EE, Green PG, Nelson DC. Mercury methylation from unexpected sources: molybdate-inhibited freshwater sediments and an iron-reducing bacterium. Appl Environ Microb. 2006;72: 457–464. doi: 10.1128/AEM.72.1.457-464.2006 16391078 PMC1352261

[37] Bodaly RA, Hecky RE, Fudge RJP. Increases in fish mercury levels in lakes flooded by the Churchill River Diversion, Northern Manitoba. Can J Fish Aquat Sci. 1984;41: 682–691.

[38] Lienesch PW, McDonald ME, Hershey AE, O’Brien WJ, Bettez ND. Effects of a whole-lake, experimental fertilization on lake trout in a small oligotrophic Arctic lake. Hydrobiologia. 2005;548: 51–66.

[39] Slotton DG, Reuter JE, Goldman CR. Mercury uptake patterns of biota in a seasonally anoxic northern California reservoir. Water Air Soil Poll. 1995;80: 841–850.

[40] Ward DM, Nislow KH, Chen CY, Folt CL. Rapid, efficient growth reduces mercury concentrations in stream-dwelling Atlantic salmon. T Am Fish Soc. 2010;139: 1–10. doi: 10.1577/T09-032.1 20436784 PMC2861578

[41] Rao ST, Ku JY, Rao KS. Analysis of toxic air contaminant data containing concentrations below the limit of detection. JAPCA J Air Waste MA. 1991;41: 442–448.

[42] Cutler DR, Edwards TC Jr., Beard KJ, Cutler A, Hess KT, Gibson J et al. Random forests for classification in ecology. Ecology. 2007;88: 2783–2792. doi: 10.1890/07-0539.1 18051647

[43] Breiman L. Random forests. Mach Learn. 2001. 45:5–32.

[44] Liaw A, Wiener M. Classification and regression by random forest. R News. 2002;2: 18–22.

[45] R Development Core Team. R: A language and environment for statistical computing. R Foundation for Statistical Computing. Vienna, Austria. 2011.

[46] Gini C. Variabilità e mutabilità. Bologna: Tipografia di Paolo Cuppini. 1912.

[47] Lepak JM, Hooten MB, Johnson BM. The influence of external subsidies on diet, growth and mercury concentrations of freshwater sport fish: implications for fisheries management and the development of fish consumption advisories. Ecotoxicology. 2012;21: 1878–1888. 22699411 10.1007/s10646-012-0921-4

[48] Stacy WL, Lepak JM. Relative influence of prey mercury concentration, prey energy density and predator sex on sport fish mercury concentrations. Sci Total Environ. 2012;437: 104–109. doi: 10.1016/j.scitotenv.2012.07.064 22922134

[49] Gaeta JW, Ahrenstorff TD, Diana JS, Fetzer WW, Jones TS, Lawson ZJ, et al. Go big or…don’t? A field-based diet evaluation of freshwater piscivore and prey size relationships. PLoS ONE. 2018. 13(3): e0194092. doi: 10.1371/journal.pone.0194092 29543856 PMC5854328

[50] Chalmers AT, Krabbenhoft DP, Van Metre PC, Nilles MA. Effects of urbanization on mercury deposition and accumulation in New England. Environ Pollut. 2014;192: 104–112. doi: 10.1016/j.envpol.2014.05.003 24907856

[51] Zhang YX, Jacob DJ, Horowitz HM, Chen L, Amos HM, Krabbenhoft DP, et al. Observed decrease in atmospheric mercury explained by global declines in anthropogenic emissions. Proc Natl Acad Sci. 2016;113: 526–531. 26729866 10.1073/pnas.1516312113 PMC4725498

[52] Lyman SN, Cheng I. Gratz LE, Weiss-Penzias P, Zhang L An updated review of atmospheric mercury. Sci Total Environ. 2020;707: 135575. doi: 10.1016/j.scitotenv.2019.135575 31784172

