# Correction: Predicting sport fish mercury contamination in heavily managed reservoirs: Implications for human and ecological health

**DOI:** 10.1371/journal.pone.0319526

**Published:** 2025-02-13

**Authors:** Jesse M. Lepak, Brett M. Johnson, Mevin B. Hooten, Brian A. Wolff, Adam G. Hansen

Table 1 is uploaded incorrectly. Please see the correct Table 1 here.

**Table 1:** Hg sample descriptions by system and species.

Reservoir	NPK	SMB	WAL	Year(s)	Individuals	Composites	Censored	Obs (ppb)
**Barr**			9	2004, 2011	9W		1W (0.3)	W (132, 298)
**Bonny**			11	2005		11W (2–5)	10W (0.1)	W (58, 106)
**Boulder City**		2		2007	2S		2S (0.1)	S (50)
**Boyd**		1	55	2006, 2011, 2012	1S, 55W		14W (0.1), 2W (0.02)	S (110), W (70, 155)
**Brush Hollow**			15	2004, 2006	5W	10W (6)	1W (0.1)	W (301, 844)
**Carter**			68	2006, 2011, 2012	68W			W (299, 425)
**Chatfield**		12	32	2004, 2013	32W	12S (5)	12S (0.3)	S (150), W (85, 198)
**Cherry Creek**			12	2007		12W (4–5)	12W (0.1)	W (50)
**Crawford**	5			2008	1N	4N (2–3)		N (198, 330)
**Douglass**			20	2007, 2012	17W	3W (5)	4W (0.02), 3W (0.1)	W (38, 96)
**Elkhead**	8	3		2005, 2007	1N	7N (4–5), 3S (3–4)		N (20, 396), S (660, 886)
**Harvey Gap**	9	4		2007, 2013	6N, 2S	3N (2), 2S (2–5)	2N, 1S (0.1)	N (166, 298), S (328, 563)
**Horseshoe**		16		2005, 2006	1S	15S (2–3)		S (420, 693)
**Horsetooth**			18	2006		18W (3–4)		W (557, 1102)
**Jackson**			8	2006		8W (4–5)		W (103, 183)
**Loveland**		2	3	2008		2S (2), 2W (4–5)	1S, 3W (0.1)	S (105), W (50)
**Lonetree**			56	2011, 2012	34W	22W (2)	2W (0.02)	W (102, 151)
**Lon Hagler**	2	2	6	2007, 2009, 2013	2N, 2S, 6W		1N (0.1)	N (88), S (241), W (61, 98)
**Martin**	9	3	4	2005	9N, 3S	4W (4)	8N, 1S, 2W (0.01)	N (30, 152), S (291), W (10)
**McPhee**	1	41	35	2004, 2010, 2012	1N, 29S, 27W	12S (5), 8W (3–5)		N (440), S (618, 816), W (339, 478)
**Narraguinnep**	4	4	25	2005, 2013	4N, 4S, 25W			N (1290), S (347), W (340, 522)
**Nee Gronda**		3	11	2005	3S, 11W		3S, 11W (0.1)	S (50), W (50)
**North Sterling**			37	2013	37W		15W (0.02)	W (45, 60)
**Pueblo**		6	10	2013	6S, 10W		1S, 8W (0.02)	S (75, 377), W (13, 43)
**Rifle Gap**	22	9	4	2007, 2008	4N, 4S, 2W	18N, 9S (2–5), 2W (4)	1N (0.1)	N (260, 440), S (585, 790), W (351)
**Sanchez**	2		12	2004	2N, 12W		2W (0.03)	N (764), W (124, 1006)
**Stagecoach**	3		2	2007		3N (2–3), 2W (3)		N (233), W (125)
**Totten**	19		3	2005	19N, 3W		15N (0.1)	N (120, 233), W (95)
**Trinidad**			18	2004, 2007		18W (2–5)	1W (0.1)	W (280, 432)
**Union**		1	11	2006	1N, 11W		1N, 10W (0.1)	S (50), W (86, 121)
**Vallecito**	23		28	2004, 2012	8N, 22W	15N, 6W (2)	11N, 2W (0.3)	N (496, 846), W (343, 819)
**Wellington**	1			2007	1N		1N (0.1)	N (50)

Each study reservoir is listed and sample sizes for northern pike (NPK), smallmouth bass (SMB), and walleye (WAL) are provided along with the year or years data were collected. Individuals that were tested for Hg concentration are indicated with a sample size followed by the letter corresponding to the first letter of the species represented. Composite samples are indicated with a sample size followed by the letter corresponding to the first letter of the species represented (N, S, and W for northern pike, smallmouth bass, and walleye, respectively) and a parenthetical range of individuals per composite sample. In systems where empirical data were not available for a given species, that system was excluded from that species analysis. Data that were censored are indicated with a sample size followed by the letter corresponding to first letter of the species represented and a parenthetical detection limit relevant to those data. Estimates of fish Hg concentrations (ppb) from empirical data (Obs) are provided following the letter corresponding to the first letter of the species represented as well as the upper 95% confidence interval (parenthetically, following the Hg concentration) when five or more samples were available to calculate error for a given system-species combination. Where error could not be calculated, only the estimated Hg concentration (ppb) is provided parenthetically without the upper 95% confidence interval.
