# Supplementary material for: Predicting sport fish mercury contamination in heavily managed reservoirs: Implications for human and ecological health
Source: PLoS One. 2023 Aug 22;18(8):e0285890. doi: 10.1371/journal.pone.0285890 (PMC10443864; doi:10.1371/journal.pone.0285890)
Supplement: S1 File — (DOCX) [file pone.0285890.s001.docx]

**Once again, we appreciate the valuable comments that the Reviewer provided. We have addressed these comments and described our responses in detail below. We hope the manuscript is now acceptable for publication. Below are our responses to Reviewer comments; our responses are provided in bold following each specific comment or set of comments.**

**REVIEWER #1:**

Line 49: Please replace “impact” with “negatively influence”. **This has been done.**

Line 144: Please replace “and” with “or”. **This has been done.**

Line 164-165: I’m still not completely satisfied with this revision and citation of Table 1. I would much prefer to see a better descriptive sentence of what Table 1 shows and why it is important that then cites Table 1 as “(Table 1)” at the end of the sentence. At this point, I will leave it to the discretion of the Editor as to whether any change is required. **We have added more detail to this sentence and now state, “Samples were collected from different systems in different years and with different methodologies (i.e., sample sizes, composites versus individuals, species analyzed) and these are described in Table 1”.**

Line 179: Please replace “norther” with “northern”. **This has been done.**

Line 382: Please delete “being the best predictors” at the end of this sentence. **This has been done.**

**As requested in the Journal Requirements. The references have been crossed checked and all those cited are reference and vice versa. We have added the 2018 to Gaeta et al. in the reference section noting it was missing. To our knowledge none of the works cited have been retracted.**
